# Safety and Effectiveness of Daratumumab in Recurrent and De Novo Focal Segmental Glomerulosclerosis After Kidney Transplant

**DOI:** 10.1016/j.xkme.2026.101384

**Published:** 2026-04-30

**Authors:** Rohan V. Mehta, Kayla B. Evans, Chintan V. Shah, Ashutosh Shukla, Amer Belal, Hisham Ibrahim, Kawther Alquadan, Muhannad Leghrouz, Georgios Vrakas, Rahul Mehta, Alfonso Santos

**Affiliations:** 1 Division of Nephrology, Hypertension and Renal Transplantation, University of Florida, Gainesville, FL; 2 University of Florida Health Transplant Center, Gainesville, FL; 3 Division of Kidney- Pancreas and Hepatobiliary Surgery, Department of Surgery, University of Florida, Gainesville, FL; 4 Division of Hospital Medicine, Department of Internal Medicine, University of Virginia, Charlottesville, VA

**Keywords:** Anti-CD38 monoclonal antibody, daratumumab, focal segmental glomerulosclerosis (FSGS), kidney transplant, plasma exchange, rituximab

## Abstract

Daratumumab, an anti-CD38 monoclonal antibody targeting plasma cells, has emerged as a potential therapy for recurrent focal segmental glomerulosclerosis (FSGS) after kidney transplantation; however, data on subcutaneous administration and its use in de novo disease are limited. We report 3 kidney transplant recipients with recurrent (n = 1) and de novo (n = 2) FSGS who were plasma exchange-dependent and/or rituximab-resistant and treated with subcutaneous daratumumab. All patients achieved remission, with sustained reductions in albuminuria and stable graft function at follow-up. Treatment was well tolerated, with no injection-related reactions; 1 patient developed transient neutropenia and oral ulcers. Subcutaneous daratumumab may represent a feasible and effective option for recurrent and de novo FSGS after kidney transplantation, including refractory cases, in conjunction with anti-CD20 therapy. Prospective studies are needed to define its role as monotherapy and the durability of response.

## Introduction

Focal segmental glomerulosclerosis (FSGS) after kidney transplantation (KT) is challenging to treat and has high morbidity and mortality. The standard of care, plasma exchange (PEX) with or without rituximab, fails in up to 43% of patients.^1^ Recently, daratumumab, a fully humanized IgG monoclonal antibody targeting CD38, has been used to treat posttransplant recurrent FSGS, achieving remission with favorable tolerability when administered intravenously.^2^ However, this agent has also been successfully used to treat multiple myeloma via the subcutaneous route, offering greater convenience and a lower incidence of infusion-related reactions.^3^ The role of subcutaneous daratumumab, whether in combination with rituximab or as monotherapy, in de novo and recurrent FSGS after KT in PEX-dependent and rituximab-resistant patients has not been described. We report 3 cases of recurrent and de novo FSGS after KT, highlighting the role of daratumumab in managing this challenging disease.

## Methods

### Case 1

**Figure 1:**
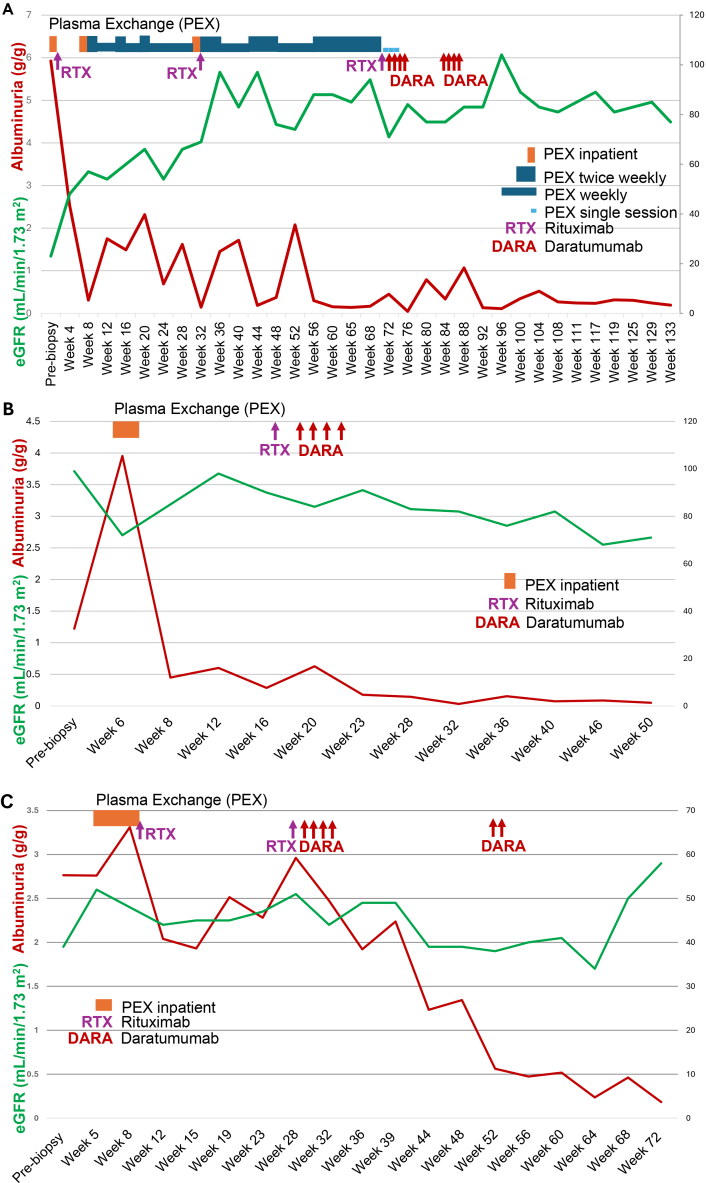
Clinical response to treatment with rituximab (RTX) and daratumumab (DARA) in cases 1, 2, and 3 (A, B, and C, respectively). The red trajectory denotes albuminuria, measured as grams of albumin per gram of creatinine (g/g), and the green trajectory represents estimated glomerular filtration rate (eGFR; mL/min/1.73 m^2^) calculated using the Chronic Kidney Disease Epidemiology Collaboration (CKD-EPI) equation. The x-axis represents time in weeks, with treatment timing (plasma exchange [PEX], RTX, and DARA) indicated at the top of each panel.

A 52-year-old South Asian woman with nongenetic primary FSGS developed biopsy-proven recurrent FSGS 7 weeks after KT (Fig 1A). She received antithymocyte globulin for induction and tacrolimus, mycophenolate, and prednisone for maintenance immunosuppression. Her initial albuminuria (urinary albumin-creatinine ratio [UACR] 6.5 g/g) responded to PEX and rituximab (375 mg/m^2^), improving to 0.36 g/g within 2 weeks after biopsy, but increased to >1 g/g 13 days after the first rituximab (375 mg/m^2^) dose. Despite an additional rituximab dose at week 31, the patient remained PEX-dependent for 74 weeks. Rituximab was repeated at week 70, followed by subcutaneous daratumumab 1,800 mg weekly for 4 doses. PEX was discontinued after the third dose, and remission, defined as UACR <0.5 g/g, lasted 5 weeks. An additional 4 doses of daratumumab were given because of rising albuminuria 12 weeks after the third rituximab dose. Adverse effects included neutropenia and oral ulcers, which resolved with granulocyte colony-stimulating factor and topical steroids, respectively.

### Case 2

A 59-year-old White man with type 2 diabetes and autosomal dominant polycystic kidney disease secondary to a *DNAJB11* mutation developed biopsy-proven de novo FSGS 52 weeks after KT (Fig 1B). He received antithymocyte globulin for induction and tacrolimus and mycophenolate for maintenance immunosuppression. After 5 PEX sessions, UACR decreased from 3.9 g/g to 0.5 g/g in 2 weeks but increased to 1 g/g by 7 weeks after PEX. Rituximab (375 mg/m^2^) was given 17 weeks after the biopsy, followed by subcutaneous daratumumab 1,800 mg/wk for 4 doses.

### Case 3

A 74-year-old White woman with autosomal dominant polycystic kidney disease because of a *DNAJB11* mutation developed biopsy-proven de novo FSGS 23 weeks after KT (Fig 1C). She received basiliximab for induction and tacrolimus and mycophenolate for maintenance immunosuppression. She underwent 10 PEX sessions and received rituximab (375 mg/m^2^) 9 weeks after biopsy, but albuminuria persisted (UACR 2.9 g/g) 19 weeks later. Rituximab was repeated at 28 weeks, followed by subcutaneous daratumumab 1,800 mg/wk for 4 doses, reducing UACR to 0.66 g/g at 50 weeks. Nineteen weeks after the last dose, 2 additional weekly doses of daratumumab were administered.

## Results

All patients also received concomitant angiotensin II receptor blockers and sodium/glucose cotransporter 2 inhibitors. Comprehensive kidney allograft biopsy findings, genetic testing results, and pertinent pre-KT clinical data of the patients are summarized in Table 1. Case 1 developed neutropenia and oral ulcers, which resolved with filgrastim and topical steroids, respectively. Overall, subcutaneous daratumumab was well tolerated, with no reported infusion reactions or injection-site events. At the time of last follow-up, case 1 remained in remission 47 weeks after the eighth dose of daratumumab (UACR 0.19 g/g; serum creatinine 0.89 mg/dL). At 12 weeks after the third rituximab dose, albuminuria worsened, necessitating an additional 4 doses of daratumumab following the initial 4-dose course, with sustained remission thereafter, suggesting successful daratumumab monotherapy. Case 2 remained in remission 28 weeks after the fourth dose (UACR 0.05 g/g; serum creatinine 1.18 mg/dL), and case 3 remained in remission 19 weeks after the sixth dose (UACR 0.18 g/g; serum creatinine 1.01 mg/dL). These results suggest a potential role for subcutaneous daratumumab in the management of FSGS after KT.

**Table 1:** Clinical, Genetic, and Allograft Biopsy Findings

Feature	Case 1	Case 2	Case 3
Native kidney biopsy	Segmental sclerosis in 2 of 10 glomeruli, including a collapsing lesion; absence of nodular sclerosis, supporting a diagnosis of primary FSGS	Nodular glomerulosclerosis consistent with diabetic nephropathy	No biopsy performed; cystic involvement of kidneys and liver
Dialysis modality	Hemodialysis	Peritoneal dialysis	Peritoneal dialysis
Dialysis duration	7 y	15 mo	12 mo
Pretransplant urine output	Absent	Preserved	Preserved
Genetic testing	Trisomy X (47,XXX); no pathogenic variants associated with FSGS identified	*DNAJB11*-associated ADPKD; no pathogenic variants associated with FSGS identified	*DNAJB11*-associated ADPKD; no pathogenic variants associated with FSGS identified
Allograft biopsy: total glomeruli	22	27	35
Globally sclerotic glomeruli	0	0	4
Segmental sclerosis	1/22	2/27	6/35
Light microscopy	Foam cells; early segmental sclerosis	Endocapillary foam cells and mononuclear cells	Foam cells, podocyte hypertrophy, adhesions
Immunofluorescence	Negative IgG, IgA, IgM, C3, C1q; albumin (1+)	IgM (1-2+), trace κ/λ; others negative	C3 (3+) mesangial; others negative
Electron microscopy	No deposits; partial FPE	No deposits; segmental FPE	Few mesangial deposits; partial FPE
Variant	FSGS; NOS	Cellular variant	FSGS; NOS

*Note:* All allograft biopsies were suggestive of FSGS, without evidence of immune complex–mediated glomerulonephritis.

Abbreviations: ADPKD, autosomal dominant polycystic kidney disease; C1q, complement component 1q; C3, complement component 3; FPE, foot process effacement; FSGS, focal segmental glomerulosclerosis; κ/λ, kappa/lambda light chains; NOS, not otherwise specified.

## Discussion

The pathogenesis of recurrent FSGS remains incompletely understood, although increasing evidence suggests that autoantibodies and plasma cells play a role.^4^ IgG autoantibodies targeting nephrin and other slit diaphragm components have been identified, and IgM deposits once thought to be passive may actively bind neoantigens such as cardiolipin on injured glomerular endothelium.^5^ This interaction can activate the classical complement pathway,^6^ leading to production of C3a, which binds to its receptor on podocytes, promoting cytoskeletal disruption and podocyte loss. Mature plasma cells, a major source of autoreactive antibodies, lack CD20 expression but strongly express CD38.^6^ Additionally, immune phenotype analyses in a prior case series have demonstrated that circulating CD38^+^ plasma cell count correlates with disease activity in FSGS.^2^^,^^7^ Therefore, daratumumab represents a compelling therapeutic option within the currently limited treatment landscape. Prior reports describing its efficacy in primary and recurrent FSGS, as well as idiopathic nephrotic syndrome, are summarized in Table 2.^2^^,^^7^^,^^8^

**Table 2:** Published Reports of Daratumumab in FSGS and Idiopathic Nephrotic Syndrome

Reference	Population	Disease and Setting	Therapy Before Daratumumab	Daratumumab Dose	Additional Therapy	Response	Key Findings
Randone et al^7^ (2024)	N = 2 (Adult = 1; Pediatric = 1)	Recurrent FSGS postkidney transplant	PEX + anakinra (case 1) PEX + RTX + anakinra (case 2)	16 mg/kg IV (multiple doses in case 1; single dose in case 2)	OBI	Partial remission (case 1); Complete remission (case 2)	Anti-CD20 + anti-CD38 therapy may be an option for recurrent FSGS refractory to conventional therapy
Angeletti et al^2^ (2024)	N = 5 (Adult)	Recurrent FSGS postkidney transplant	PEX alone (cases 1, 2, and 5) PEX + RTX (cases 3 and 4)	16 mg/kg IV	RTX	Complete remission	CD38^+^ plasma cells correlate with disease activity; supports targeting anti-CD20 + anti-CD38 therapy as a pilot approach
Naciri Bennani et al^8^ (2025)	N = 4 (Adult = 2; Pediatric = 2)	Idiopathic nephrotic syndrome (native = 3; postkidney transplant = 1)	OBI (case 1) IA/DFPP + OBI (cases 2 and 3) IA + RTX (case 4)	500 mg IV (case 1) 1,000 mg/1.73 m^2^ IV (case 2)	OBI or RTX	Complete remission	Potential for anti-CD20 + anti-CD38 in both native and after transplant INS
				1,000 mg IV then 1800 mg SQ × 3 (Case 3)			
				1,800 mg SQ × 3 (Case 4)			

Abbreviations: DFPP, double-filtration plasmapheresis; FSGS, focal segmental glomerulosclerosis; IA, immunoadsorption; INS, idiopathic nephrotic syndrome; IV, intravenous; OBI, obinutuzumab; PEX, plasma exchange; RTX, rituximab; SQ, subcutaneous.

In this report, we demonstrate, to our knowledge, for the first time, beneficial effects of daratumumab in de novo FSGS after KT. The effectiveness of anti-plasma cell therapy in recurrent and de novo FSGS after KT warrants further study to determine whether similar immunologic mechanisms and therapeutic effects can be observed in idiopathic FSGS affecting native kidneys, particularly in steroid- and/or rituximab-resistant disease.^8^

When daratumumab is administered intravenously, infusion-related reactions are common, occurring in approximately 50% of patients.^3^ Consequently, infusion times are prolonged, with a median of approximately 7 hours, often necessitating a split-dose strategy. The PAVO trial was a phase 1b multicenter trial evaluating daratumumab formulated with recombinant human hyaluronidase PH20 to enable subcutaneous administration. Pharmacokinetic analyses demonstrated that a 1,800 mg subcutaneous daratumumab regimen achieves exposures comparable with those of the 16 mg/kg intravenous regimen used in multiple myeloma.^3^ The most common adverse effects associated with this subcutaneous dose included hematologic toxicities, upper respiratory tract infection, pyrexia, and diarrhea. The rate of infusion-related reactions was 24.4%, representing a significant improvement over intravenous infusion, and injection-site reactions such as erythema, induration, pain, and paresthesia were mild and self-limited.^3^ Because prior reports of daratumumab in most glomerular diseases have almost exclusively used intravenous administration, our series, to our knowledge, is the first to employ subcutaneous administration of daratumumab in PEX- and rituximab-resistant FSGS, leveraging the pharmacokinetic equivalence and improved tolerability to ease administration and improve patient convenience.

Interpretation of our findings is limited by the retrospective, uncontrolled design; however, spontaneous remission in FSGS after KT is rare, supporting the likelihood of a true therapeutic effect. The marked benefit observed in rituximab-resistant patients suggests that anti-CD38 therapy may offer meaningful efficacy, although whether daratumumab monotherapy can be used in rituximab-refractory FSGS remains to be determined. Several other limitations should be acknowledged. Serial immune phenotyping was not available, limiting our ability to distinguish the independent effects of anti-CD38 therapy from residual or synergistic anti-CD20 activity.

In conclusion, this case series demonstrates that subcutaneous daratumumab is a feasible, well tolerated, and potentially effective therapy for both recurrent and de novo FSGS after KT, including patients refractory to PEX and rituximab. These sustained remissions reinforce the pathogenic contribution of CD38^+^ plasma cells in FSGS, suggesting that daratumumab monotherapy merits consideration. Subcutaneous administration offers practical advantages over intravenous therapy without compromising efficacy. Prospective, controlled studies are needed to define its optimal role, the durability of response, and its applicability to FSGS in native kidneys as well as in de novo and recurrent FSGS after KT. Emerging data suggest that obinutuzumab may offer more effective B-cell depletion than rituximab, with small series showing efficacy in refractory nephrotic syndrome and recurrent FSGS, including rituximab-resistant cases.^7^^,^^9^ Combining B-cell and plasma cell targeting with obinutuzumab and daratumumab has also been linked to sustained remission in severe posttransplant recurrence.^7^ However, evidence remains limited, and its role relative to rituximab or in combination strategies needs further investigation.
